# Comparative Metagenomics of the Polymicrobial Black Band Disease of Corals

**DOI:** 10.3389/fmicb.2017.00618

**Published:** 2017-04-18

**Authors:** Julie L. Meyer, Valerie J. Paul, Laurie J. Raymundo, Max Teplitski

**Affiliations:** ^1^Soil and Water Science Department, University of Florida-Institute of Food and Agricultural SciencesGainesville, FL, USA; ^2^Smithsonian Marine StationFort Pierce, FL, USA; ^3^University of Guam Marine LaboratoryMangilao, Guam

**Keywords:** coral microbiology, coral disease, cyanobacteria, microbiome, *Roseofilum reptotaenium*

## Abstract

Black Band Disease (BBD), the destructive microbial consortium dominated by the cyanobacterium *Roseofilum reptotaenium*, affects corals worldwide. While the taxonomic composition of BBD consortia has been well-characterized, substantially less is known about its functional repertoire. We sequenced the metagenomes of Caribbean and Pacific black band mats and cultured *Roseofilum* and obtained five metagenome-assembled genomes (MAGs) of *Roseofilum*, nine of Proteobacteria, and 12 of Bacteroidetes. Genomic content analysis suggests that *Roseofilum* is a source of organic carbon and nitrogen, as well as natural products that may influence interactions between microbes. Proteobacteria and Bacteroidetes members of the disease consortium are suited to the degradation of amino acids, proteins, and carbohydrates. The accumulation of sulfide underneath the black band mat, in part due to a lack of sulfur oxidizers, contributes to the lethality of the disease. The presence of sulfide:quinone oxidoreductase genes in all five *Roseofilum* MAGs and in the MAGs of several heterotrophs demonstrates that resistance to sulfide is an important characteristic for members of the BBD consortium.

## Introduction

Black Band Disease (BBD) is a globally distributed coral disease that devastates dozens of species of corals, including large, reef-building scleractinians (Sato et al., 2016). It is easily recognized by the appearance of a dense dark purple or black band, which is the visible accumulation of phycoerythrin-rich filamentous cyanobacteria. In the Caribbean, known as a “hotbed” of coral disease (Weil et al., 2006), BBD is more prevalent in warmer, shallower waters (Kuta and Richardson, 2002), and manipulative experiments have demonstrated that BBD progresses faster at higher light levels and temperature (Kuehl et al., 2011; Sato et al., 2011). The cyanobacterium recently renamed *Roseofilum reptotaenium* has been implicated as the causative agent of BBD within the polymicrobial disease consortium (Casamatta et al., 2012). Strains of *Roseofilum* have been cultivated in the laboratory, but like other filamentous cyanobacteria, *Roseofilum* cannot be fully isolated, only grown in non-axenic, unicyanobacterial cultures (Richardson et al., 2014). This conserved interdependence of *Roseofilum* and heterotrophic bacteria may be linked to metabolic requirements and may explain why this cyanobacterium is found within apparently healthy coral microbiomes (Meyer et al., 2016). In many ways, BBD consortia resemble microbial mats found in tropical lagoons (Echenique-Subiabre et al., 2015), mangroves (Guidi-Rotani et al., 2014), and modern marine stromatolites (Ruvindy et al., 2016) that are characterized by steep physicochemical gradients and the presence of Cyanobacteria, Bacteroidetes, and Proteobacteria (especially Alpha-, Delta-, and Gammaproteobacteria; Bolhuis et al., 2014). With the large size of filamentous cyanobacteria relative to other bacterial cells, their production of exopolysaccharides that consolidate the mat, and their critical roles in converting inorganic carbon, nitrogen, and sulfur to organic forms, Cyanobacteria are the pioneering microorganisms of microbial mats (Bolhuis et al., 2014). Likewise, *Roseofilum* is the engineer of the black band consortium, creating a narrow polymicrobial band on the surface of reef-building corals (Miller and Richardson, 2011; Casamatta et al., 2012; Richardson et al., 2014).

While the composition of BBD has been studied for decades (Miller and Richardson, 2011), the recent application of high-throughput sequencing to characterize the microbial community structure in both the black band layer and adjacent healthy coral epimicrobiome has uncovered two important factors in understanding this disease. First, BBD is highly localized, and in as little as 10 cm from the leading edge of the black band mat, the microbial community in the surface mucus layer is indistinguishable from that of healthy corals (Meyer et al., 2016). Second, *Roseofilum* is a rare but ubiquitous member of healthy Caribbean coral microbiota, implying that growth of *Roseofilum* is constrained in healthy tissue until undefined restrictions are removed (Meyer et al., 2016). Together, this suggests that while *Roseofilum* is capable of engineering the highly altered black band layer, its influence is spatially limited and its pathogenesis is contextual. To uncover the mechanisms through which *Roseofilum* proliferates and engineers a new environment on the surface of corals during BBD, we examined the metagenomic potential of members of black band consortia *in situ* and *in vitro*.

## Methods

Samples of coral surface microbiota and the BBD mat were collected by aspiration with sterile, needle-less syringes from the Florida Keys, Belize, Honduras, and Guam (Table S1), as in Meyer et al. (2016).

### Enrichment culturing of the black band disease consortium

The black band layer of an infected *Montastraea cavernosa* coral was collected at Looe Key Reef, Florida on April 23, 2014 and used to isolate Cyanobacteria from the disease consortium. *Roseofilum* was grown in unicyanobacterial, but not axenic cultures, in a medium containing four parts artificial seawater made from 36 g/L Red Sea Coral Pro Salts and 1 part Cyanobacterial BG-11 media (ATCC Medium 616), at pH 7, room temperature, with 12 h of light and dark per day. Cyanobacterial cultures were confirmed as unicyanobacterial by amplifying extracted community DNA with cyanobacterial specific 16S rRNA primers (Nübel et al., 1997) and directly sequencing the cleaned PCR product by Sanger sequencing at the DNA Lab of Arizona State University (GenBank Accession No. KP689103).

### 16S illumina tag sequencing

Genomic DNA for 16S Illumina tag sequencing was extracted with a PowerSoil DNA isolation kit (MoBio, Carlsbad, CA). The V6 region of 16S rRNA genes was amplified in triplicate for each sample with previously reported Illumina-compatible primers designed for bacteria (Eren et al., 2013), using previously described conditions for amplicon library preparation and analysis (Meyer et al., 2016). Sequencing of amplicon libraries was performed at the Genomics Core Facility at Pennsylvania State University on an Illumina MiSeq with a 150-bp paired-end protocol, using single indexing. Sequencing reads are publicly available through NCBI's Sequence Read Archive (SRA) under the BioProject ID PRJNA269585. Analysis of the 16S amplicon libraries was performed using the previously described parameters (Meyer et al., 2016). Briefly, sequences were clustered at 97% using the subsampled open reference OTU picking method (Rideout et al., 2014) with no removal of singletons in Qiime v.1.8 (Caporaso et al., 2010), and assigned taxonomy with the Greengenes database v.13.8 (DeSantis et al., 2006). Community structure was analyzed in R with Phyloseq (McMurdie and Holmes, 2013) and plotted with ggplot2 (Wickham, 2009).

### Metagenomic library construction and sequencing

For metagenomic library construction, DNA and RNA were simultaneously extracted from the BBD samples from infected corals using a Qiagen AllPrep DNA/RNA Micro Kit (Germantown, MD) from the Belize and Florida samples. Only DNA was extracted from the Guam sample due to limited biomass using a PowerSoil DNA isolation kit (MoBio, Carlsbad, CA). Metagenomic libraries were constructed with a TruSeq DNA Sample Preparation Kit (Illumina, San Diego, CA) and sequenced at the University of Maryland Institute for Bioscience and Biotechnology Research on an Illumina HiSeq with a 100-bp paired-end protocol.

### Metagenomic data analysis

Sequencing reads were quality-filtered by trimming adaptors with cutadapt (Martin, 2011) and filtering reads for a minimum quality score of 30, minimum length of 100 bp, and discarding all sequences with ambiguous base calls using Sickle (Joshi and Fass, 2011), and interlaced with the shuffleSequences_fastq.pl script from velvet (Zerbino and Birney, 2008) prior to assembly. The unassembled, quality-filtered reads are publicly available through NCBI's Sequence Read Archive (SRA) under the BioProject ID PRJNA269585. Metagenomes were assembled individually with IDBA-UD (Peng et al., 2012) with k-mer sizes of 30–80 and submitted to IMG-MER (Markowitz et al., 2006) for annotation. Annotated metagenomic assemblies are publicly available in IMG under the IMG Genome IDs 3300003272, 3300003311, 3300003317, 3300003641, 3300003309 (Table S2). Unassembled reads were mapped to the assembled contigs with bowtie2 (Langmead and Salzberg, 2012) and alignment statistics were recovered with samtools (Li et al., 2009). The metagenomic sequencing coverage was assessed with non-pareil (Rodriguez and Konstantinidis, 2014).

Metagenome-assembled genomes (MAGs) were retrieved from the assembled metagenomic contigs by binning based on tetranucleotide frequencies using Emergent Self-Organizing Maps (ESOM) as in Dick et al. (2009), using the protocol described at https://github.com/tetramerFreqs/Binning. The training algorithm was K-batch, the starting radius was 50, and the map sizes were as follows: BLZ4 181 rows × 362 columns, BLZD 144 × 288, Cyano 244 × 488, LKpool 199 × 398, and Guam 180 × 360. Within each MAG, any scaffolds with unusually high or low coverage of mapped reads were removed from the bin. Summary statistics for each MAG were determined with QUAST v. 2.3 (Gurevich et al., 2013). Genome completeness and the presence of contamination within MAGs was determined with Anvi'o v. 2.1.0 (Eren et al., 2015), using four different sets of single-copy genes. Antibiotic and secondary metabolite biosynthetic genes in MAGs were identified using antiSMASH v. 3.0 (Weber et al., 2015). The two-way average nucleotide identity (ANI) between closely related genomes was calculated as in Goris et al. (2007) through the online tools of the Environmental Microbial Genomics Laboratory (enve-omics lab) at the Georgia Institute of Technology. MAGs were submitted to both IMG-ER (Markowitz et al., 2006) and RAST (Aziz et al., 2008) for annotation and the IMG-annotated MAGs are publically available under the accession numbers listed in Table 1. Statistical analyses of SEED subsystems annotation from RAST profiles of MAGs were performed with STAMP (Parks et al., 2014), using Storey FDR multiple test correction, *q* < 0.05 and effect size >0.6. Summary figures were created in R with ggplot2. Pan-genome analysis of *Roseofilum* MAGs was performed with Roary v.3.4.1 (Page et al., 2015). A second pan-genome analysis of the five *Roseofilum* MAGs described here, along with the recently published MAGs of *Roseofilum* from the Great Barrier Reef (Sato et al., 2016), a *Roseofilum* culture from the Great Barrier Reef (Buerger et al., 2016), and a *Geitlerinema* culture from Caribbean black band mat (Den Uyl et al., 2016) was performed with Anvi'o v.2.1.0, in which proteins were clustered with the MCL algorithm (van Dongen and Abreu-Goodger, 2012) using 2 for the MCL inflation parameter and identified by NCBI's blastp (Altschul et al., 1990).

**Table 1:** **Characteristics of metagenome-assembled genomes from Black Band Disease mats and cultured *Roseofilum reptotaenium***.

**Binned genome name**	**IMG ID**	**Avg. coverage**	**No. of contigs**	**Largest contig (bp)**	**Total length (Mbp)**	**% G+C**	**% Complete**	**% Redundancy**
Roseofilum_Cyano_bin5	2627853559	475	177	146,729	4.54	44.96	82	3
Roseofilum_LKpool_bin4	2627853563	16	753	16,752	3.36	44.26	49	2
Roseofilum_BLZ4_bin2	2627853561	135	281	144,531	5.23	44.95	95	3
Roseofilum_BLZD_bin1	2627853562	109	233	111,190	5.36	44.97	96	3
Roseofilum_Guam_bin12	2627853560	776	126	157,365	5.45	44.81	98	4
Bacteroidetes_Cyano_bin6	2627853567	41	26	557,448	3.09	48.91	81	0
Bacteroidetes_Cyano_bin10	2627853572	16	344	113,992	7.0	46.02	83	2
Bacteroidetes_Cyano_bin11	2627853566	28	17	904,008	3.87	34.13	99	2
Bacteroidetes_LKpool_bin1	2627853570	50	201	192,017	4.80	34.18	60	2
Bacteroidetes_LKpool_bin3	2627853577	14	307	10,892	1.08	51.56	28	0.7
Bacteroidetes_LKpool_bin7	2627853568	135	331	157,835	4.63	34.56	94	9
Bacteroidetes_BLZ4_bin4	2627853578	80	119	348,523	4.53	34.31	97	1
Bacteroidetes_Guam_bin1	2627853571	27	258	107,852	3.98	38.73	71	1
Bacteroidetes_Guam_bin2	2627853573	11	167	8,570	6.14	38.94	35	0
Bacteroidetes_Guam_bin4	2627853576	10	336	9,241	1.25	45.51	35	0
Bacteroidetes_Guam_bin8	2627853574	16	197	137,588	3.31	37.45	53	0
Bacteroidetes_Guam_bin9	2627853575	11	638	18,297	2.43	38.83	37	9
Alphaproteobacterium_Cyano_bin1	2627853580	13	535	29,727	3.35	51.18	54	0.7
Gammaproteobacterium_Cyano_bin9	2627853583	20	227	84,639	3.12	58.67	71	0
Desulfovibrio_LKpool_bin5	2627853564	17	503	15,593	2.27	46.90	50	2
Alteromonadales_BLZ4_bin3	2627853581	23	372	62,452	4.05	42.04	78	0.7
Desulfovibrio_BLZ4_bin6	2627853565	18	541	23197	3.15	47.37	73	3
Rhodospirillales_BLZ4_bin7	2627853579	35	607	42,843	4.46	48.32	98	4
Oceanospirillum_Guam_bin7	2627853582	11	477	35,999	2.02	47.06	54	2
Alteromonadales_Guam_bin6	2627853584	10	321	8,646	1.11	45.35	7	0.7
Vibrio_Guam_bin5	2627853569	9	237	10,868	0.82	44.84	21	0

## Results and discussion

### Microbial community structure from 16S rRNA gene sequencing

**Figure 1:**
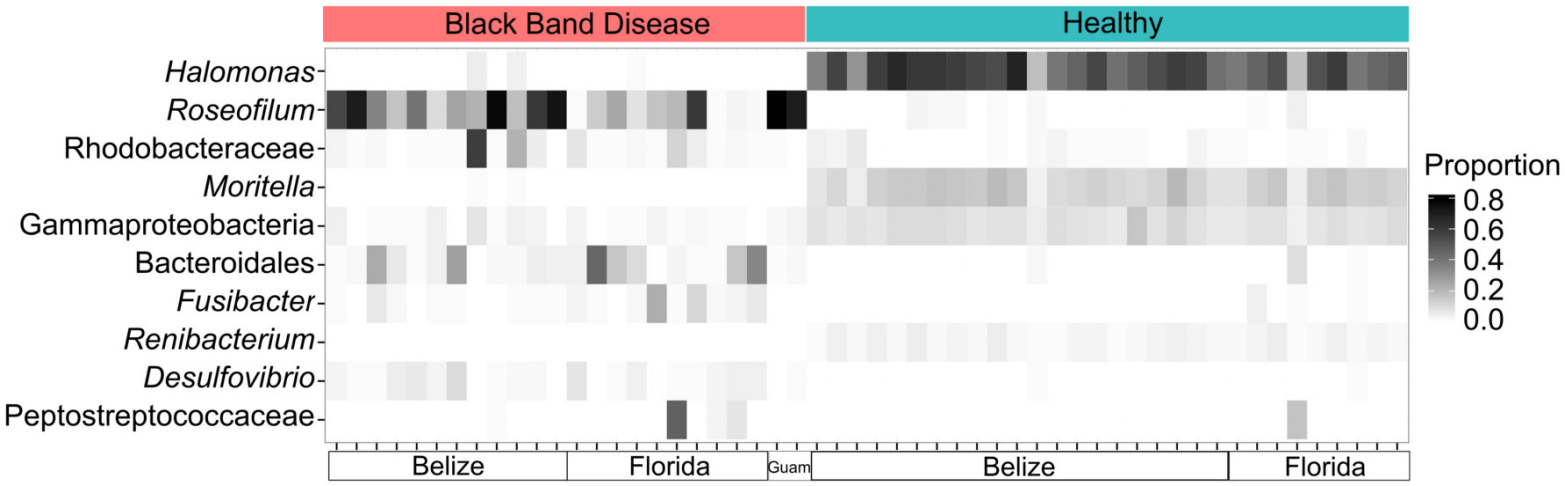
**Community shifts in Black Band Disease (BBD) consortia and healthy coral epimicrobiomes**. Relative abundance of the 10 most common bacterial genera in 16S rRNA gene libraries from 54 coral surfaces. Each column in the heatmap represents an individual coral surface microbiome sample.

Bacterial community structure was determined for 24 BBD consortia, 30 healthy coral surface microbiomes, and one *Roseofilum* culture by amplification and Illumina sequencing of the V6 hypervariable region of 16S rRNA genes (Table S1). Consistent with previous results from Caribbean corals only (Meyer et al., 2016), the reanalysis of the bacterial community structure with both Caribbean and Pacific corals demonstrates the prevalence of *Roseofilum*, Rhodobacteraceae, Bacteroidales, and *Desulfovibrio* in black band consortia in contrast to the high proportions of Gammaproteobacteria (such as *Halomonas* and *Moritella*) and Actinobacteria (such as *Renibacterium*) in healthy epimicrobiomes (Figure 1). Comparison of the composition of 24 *in situ* BBD samples and a unicyanobacterial, but not axenic *Roseofilum* culture revealed that three proteobacterial genera were depleted in the culture. The relative abundance of *Vibrio* in the culture was two orders of magnitude lower (0.001% of reads in the *Roseofilum* culture vs. ~0.1% in BBD), *Arcobacter* was three orders of magnitude lower (0.0004% vs. ~0.1%), and *Desulfovibrio* was three orders of magnitude lower (0.0002% vs. ~0.01%).

The remarkable degree of conservation of taxa in the *Roseofilum* culture compared to *in situ* BBD metagenomes suggests a strong interdependence of members of the disease consortium that is unrelated to the coral host. Specifically, no major BBD taxonomic groups were entirely missing in the *Roseofilum* culture and the proportions of Bacteroidetes, Deltaproteobacteria, and Gammaproteobacteria were relatively unchanged between the *Roseofilum* culture and *in situ* BBD. However, three proteobacterial genera (*Vibrio, Arcobacter*, and *Desulfovibrio*) were detected in lower relative abundance in the culture compared to *in situ* BBD, based on 16S rDNA amplicon libraries. Although these genera may not be the most abundant organisms within the BBD consortium, each has the potential to impact the progression of the disease, as both *Vibrio* and *Arcobacter* are genera containing pathogens and both *Arcobacter* and *Desulfovibrio* are involved in sulfur cycling. Recent metatranscriptomic analysis of BBD consortia in the Red Sea demonstrated that the most highly expressed functional gene among BBD vibrios was a thiamin transporter (Arotsker et al., 2016), suggesting that *Roseofilum*, as a producer of thiamin, has the capacity to encourage the growth of vibrios that lyse coral tissue (Arotsker et al., 2009) during the colonization of adjacent healthy tissue. However, interactions between *Roseofilum* and vibrios are likely complex, as we previously demonstrated mutual exclusion between *Roseofilum* and *Vibrio* spp., that may be mediated through production of lyngbic acid by *Roseofilum* that is capable of disrupting the CAI-1 vibrio quorum-sensing pathway (Meyer et al., 2016). While previous work revealed a signficant co-occurrence of *Roseofilum* and *Desulfovibrio* in BBD consortia (Meyer et al., 2016), *Desulfovibrio* was not co-enriched with *Roseofilum* in the culture. This indicates that the co-occurrence of these two genera is related to opportunistic interactions on the coral surface, rather than a specific interactive relationship. Furthermore, it suggests the two groups contribute individually to disease progression.

### Assembled metagenomes and metagenome-assembled genomes from black band disease consortia

Four metagenomes of the disease consortia were generated from the black band community on *Pseudodiploria strigosa* and *Orbicella annularis* corals from Belize, *Goniopora fruticosa* from Guam, and a pooled sample from *M. cavernosa* and *O. faveolata* from Florida. A fifth metagenome was generated from a unicyanobacterial, but not axenic, culture of *R. reptotaenium* isolated from a black band layer on *M*. *cavernosa* collected in Florida. Each metagenomic dataset contained 16,853,081–44,199,247 read pairs after quality filtering (Table S2). Annotated metagenomic assemblies contained 69,548–612,757 protein-coding genes (Table S2). The coverage of metagenomic sequencing was assessed with non-pareil using the unassembled quality-filtered sequencing reads and ranged from 87 to 99%, indicating that this sequencing depth was adequate to capture most of the unique sequences in the extracted community DNA, even if not all of this diversity was captured in the metagenomic assemblies. To assess the quality of the metagenomic assemblies, the unassembled sequencing reads were mapped to the metagenomic assemblies, with an overall alignment rate that ranged from 41% (BLZD) to 92% (Cyano), where a higher alignment rate indicates that the metagenomic assembly encompassed more of the diversity present in the sequencing reads (Table S2). Between 2 and 24 unique genomes were detected within each metagenomic assembly (Table S2) by Anvi'o (Eren et al., 2015), using four different databases of single copy genes as in Delmont and Eren (2016).

The number of protein-coding genes in each metagenome ranged from 69,548 to 612,757 (Table S2). Of the annotated genes in the assembled metagenomes, 12 to 47% could be assigned to bacterial phyla (16,674–79,576 bacterial genes per metagenome). Less than 1% of annotated genes in any metagenomic assembly could be assigned to archaeal phyla. The remaining genes were either unassigned or identified as eukaryotic. A total of 53 bacterial phyla and 6 archaeal phyla were identified in the metagenomic assemblies (Table S3). The four most abundant bacterial phyla (Cyanobacteria, Bacteroidetes, Proteobacteria, and Firmicutes) constituted 80–95% of the annotated bacterial genes in the four environmental metagenomic assemblies (Figure S1). In the *Roseofilum* culture, these four phyla constituted 68% of annotated genes with taxonomic assignment, and Planctomycetes were more abundant than Firmicutes (Figure S1). The proportions of functional genes assigned to Cyanobacteria and Bacteroidetes in the metagenomic assemblies matched the proportion of these phyla detected by 16S rRNA gene surveys, while the proportion of Proteobacteria was higher in 16S gene surveys than in metagenomic assemblies (Figure S1).

Binning of the assembled metagenomic datasets produced a total of 26 MAGs, with estimated genome coverage ranging from 9 × to 776 × (Table 1). The annotated MAGs are publicly available in IMG and under the accession numbers listed in Table 1. MAGs had varying levels of estimated genome completeness and redundancy (Table 1). With the exception of the LKpool *Roseofilum*, which was assembled from pooled coral hosts, the MAGs of *Roseofilum* were relatively complete and contained low redundancy (4% or less of the single-copy genes in the *Roseofilum* MAGs were present in more than one copy). Only one cyanobacterial MAG and one full-length cyanobacterial 16S rRNA gene were retrieved from each assembled metagenome. To corroborate the low diversity of cyanobacterial genomes, contigs identified as containing cyanobacterial genes based on IMG annotation from each of the five metagenomic assemblies were pooled *in silico* and analyzed for the number of genomes present. Assembled contigs annotated as Cyanobacteria from the Belize *Orbicella* metagenome (BLZ4) contained up to three copies of single copy genes, while cyanobacterial contigs from the other metagenomes contained one to two copies of single copy genes, indicating a low diversity of Cyanobacteria in the black band layer. The cyanobacterial 16S rRNA gene sequence was identical in metagenomic assemblies from the three Caribbean samples and the unicyanobacterial culture, while minor variation (98.9% similarity) was detected in the cyanobacterial 16S rRNA gene sequence from Guam. The 16S rRNA gene sequences recovered from the Caribbean cyanobacterial MAGs are identical to the reported sequence in *R. reptotaenium* (EU743965) with the exception of one nucleotide at the 5′ end of the published sequence for the type species. The two-way average nucleotide identity of the four Caribbean *Roseofilum* MAGs was >99% similarity, while the Guam *Roseofilum* MAG showed around 94% similarity to the Caribbean *Roseofilum* MAGs. In addition to the five *Roseofilum* MAGs, MAGs of 12 Bacteroidetes, and 9 Proteobacteria were recovered by binning, including an unclassified gammaproteobacterium, Bacteroidales, and *Desulfovibrio*, which have been detected as common in BBD consortia through 16S amplicon surveys (Figure 1; Miller and Richardson, 2011; Meyer et al., 2016).

### Functional characteristics of black band disease metagenomes and metagenome-assembled genomes

**Figure 2:**
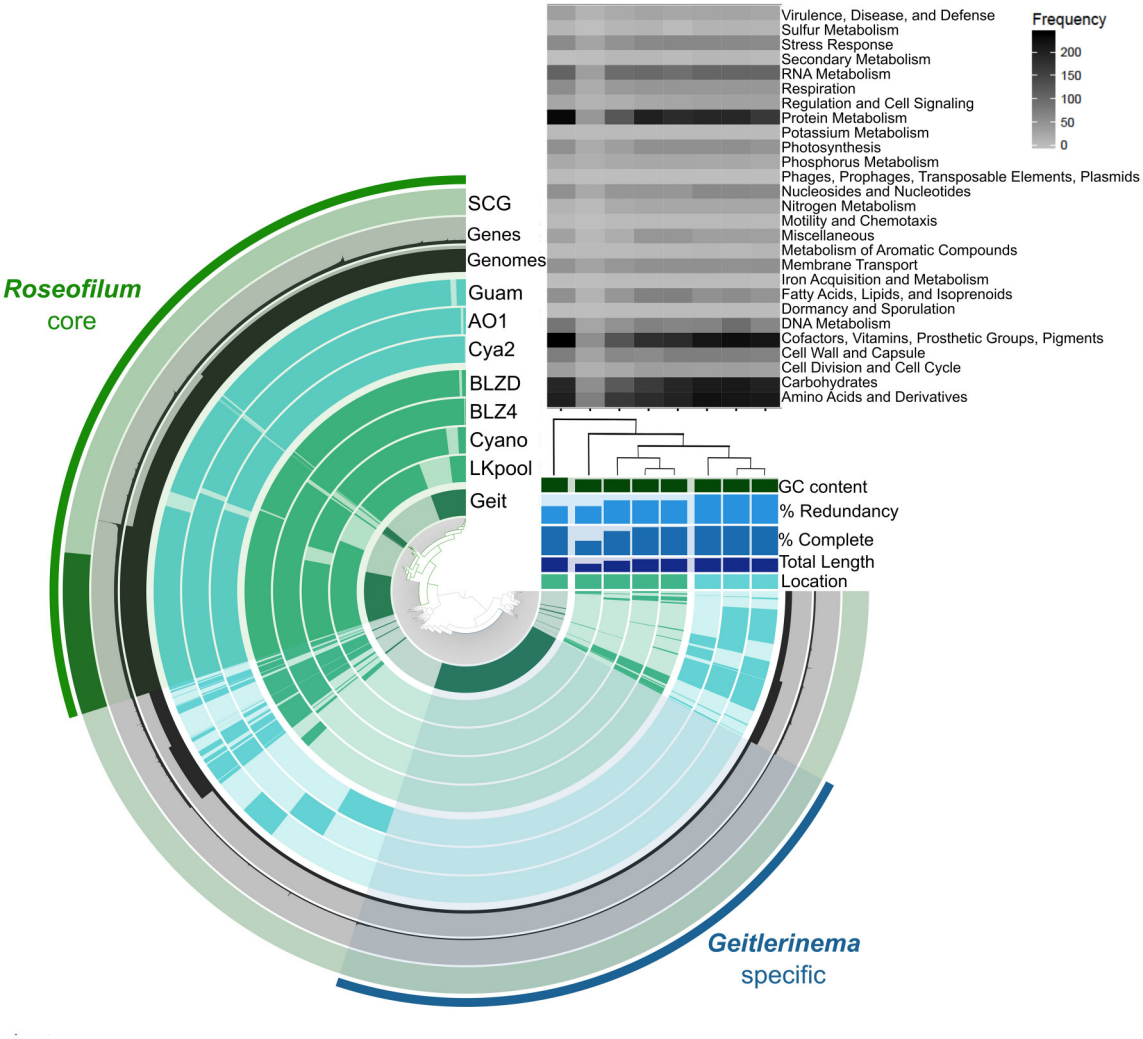
***Roseofilum*** **pan-genome**. Protein clusters from seven *Roseofilum* and one *Geitlerinema* metagenome-assembled genomes (MAGs) are displayed in the central dendrogram and genes present in each MAG are indicated on concentric rings. The outer-most ring displays Single Copy Genes present in the *Roseofilum* core genome. The next two rings display the number of genes and number of MAGs, respectively, for each protein cluster. Upper right: Relative abundance of genes in each MAG assigned to Level 1 SEED subsystems annotation. The % completeness and % redundancy of each MAG is based on the presence/absence of single-copy genes. Clustering of MAGs (right) was based on the relative abundance of proteins and reflected the location: Green for Caribbean samples and Teal for Pacific samples. “Geit” is the cultured *Geitlerinema* sp. BBD 1991 MAG isolated from *Orbicella annularis* in Florida (Den Uyl et al., 2016), “LKpool” is the *Roseofilum* MAG from a pooled sample from *Montastraea cavernosa* and *O. faveolata* from Florida, “Cyano” is the cultured *Roseofilum* MAG isolated from *M*. *cavernosa* collected in Florida, “BLZ4” is the *Roseofilum* MAG from *Orbicella annularis* in Belize, “BLZD” is the *Roseofilum* MAG from *Pseudodiploria strigosa* in Belize, “Cya2” is the *Roseofilum* MAG from *Montipora hispida* on the central Great Barrier Reef (Sato et al., 2016), “AO1” is the cultured *Roseofilum* MAG isolated from *Pavona duerdeni* on the central Great Barrier Reef (Buerger et al., 2016), and “Guam” is the *Roseofilum* MAG from *Goniopora fruticosa* in Guam.

The five *Roseofilum* MAGs had similar functional profiles based on KEGG Ontology (Figure S2), as well as similar gene synteny (Figure S3) in contrast to the more taxonomically and functionally diverse Bacteroidetes and Proteobacteria MAGs (Figure S2). Pan-genomic clustering of protein-coding genes in *Roseofilum* and *Geitlernema* MAGs (Figure 2) reveals a conserved core of *Roseofilum* functional genes that is distinct from the *Geitlerinema* MAG based on sequence variation, as expected for taxa assigned to different genera. However, the overall functional profile of all eight MAGs, based on SEED subsystems, is well-conserved (with the exception of the less-complete LKpool *Roseofilum*; Figure 2). Clustering of *Roseofilum* MAGs based on the relative abundance of proteins showed a clear geographic signal, with Caribbean MAGs clustering together and Pacific MAGs (from three different research groups) clustering together (Figure 2). A total of 36,649 genes in 8,690 protein clusters were identified in the eight MAGs. The *Roseofilum* core indicated in Figure 2 contained 27,153 genes in 3,470 protein clusters, while the *Geitlerinema* specific bin contained 2,702 genes. Clusters in the *Roseofilum* core were defined as being present in at least five of the seven *Roseofilum* MAGs. The remaining, less-conserved regions contained a total of 6,794 genes in 2,634 protein clusters. Of these less-conserved protein clusters, 40% (1055 clusters) contained only one gene and 18% (485 clusters) contained only two genes.

**Figure 3:**
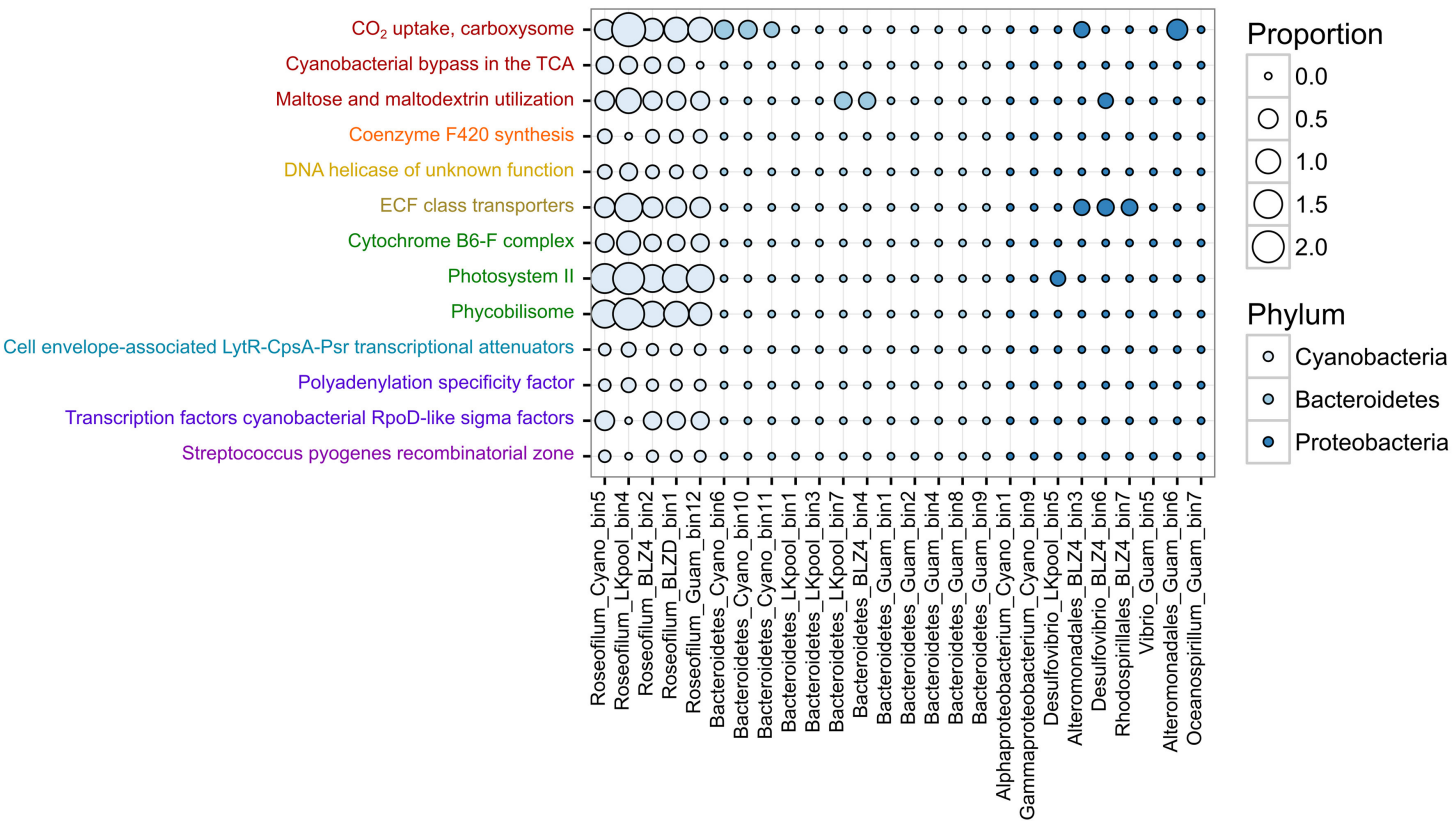
**Functional differences in metagenome-assembled genomes from Black Band Disease consortia**. Differences in the relative abundance of Level 3 SEED subsystems annotations among metagenome-assembled genomes from three phyla. Functional annotations with the same lettering color belong to the same Level 1 SEED subsystems category. Only features passing the filtering criteria are shown (*q* < 0.05, effect size > 0.6, multiple test correction with Storey FDR).

To determine the potential roles and interactions between different members of the polymicrobial disease consortium, we tested for differences in the relative abundances of protein-coding genes assigned to SEED subsystems (Overbeek et al., 2014) level 3 functions. Examination of differentially represented functions among the three predominant phyla (Cyanobacteria, Bacteroidetes, Proteobacteria) revealed 87 subsystems with statistically different abundances (Table S3). Of these differentially represented functions, 13 had an effect size >0.6, meaning that the difference in the mean relative abundance was >60% and encompassed mostly functions that were present solely in the *Roseofilum* MAGs and not in the MAGs of heterotrophs (Figure 3). Genes associated with carbon fixation (Subsystem CO_2_ uptake, carboxysome), micronutrient acquisition (Subsystem ECF class transporters), and phototrophy (Subsystems Cytochrome B6-F complex, Photosystem II, and Phycobilisome) were more abundant in the *Roseofilum* MAGs than the MAGs of heterotrophs (Figure 3). In addition, functions related to carbon respiration (Subsystems Cyanobacterial bypass in the TCA, Maltose and maltodextrin utilization), and the redox-regulated molecular chaperone Hsp33 (Subsystem Steptococcus pyogenes recombinatorial Zone) were primarily unique to the *Roseofilum* MAGs (Figure 3).

Differentially abundant functions between the three phyla also suggest a strong internal cycling of organic nitrogen within the black band layer in which Bacteroidetes and Proteobacteria degrade amino acids to produce urea and urea is degraded by *Roseofilum*. Both Bacteroidetes and Proteobacteria MAGs contained genes for the degradation of branched chain amino acids (leucine, isoleucine, valine) that were not present in the five *Roseofilum* MAGs, and the *Roseofilum* MAGs had more genes associated with the degradation of urea than Bacteroidetes and Proteobacteria MAGs (Table S4). In addition, genes for the production and degradation of cyanophycin, a polypeptide-like nitrogen storage compound, were present in the five *Roseofilum* MAGs and in the Cyano_bin9_Gammaproteobacteria MAG (Table S5). Genes for the assimilation of ammonia and nitrate were common in the MAGs of both *Roseofilum* and heterotrophs, demonstrating that each member of the consortium utilizes multiple pathways to assimilate nitrogen in the nitrogen-limited coral reef ecosystem. Genes for nitrogen fixation (*nifHDK*) were detected in the five *Roseofilum* MAGs as well as in two Proteobacteria MAGs (BLZ4_bin3_Alteromonadales, BLZ4_bin7_Rhodospirillales; Table S5). The diversity of methods utilized by *Roseofilum* for the acquisition of nitrogen mirrors those found in the *Geitlerinema* strain isolated from BBD (Den Uyl et al., 2016).

The multiple, diverse Bacteroidetes MAGs recovered here from both Caribbean and Pacific BBD underscore the previously underappreciated predominance of Bacteroidetes in BBD. While Bacteroidetes have been detected in 16S rRNA gene surveys of BBD and have been hypothesized to play a role in the pathogenesis of BBD (Frias-Lopez et al., 2004), the abundance of Bacteroidetes MAGs and high relative abundance of genes assigned to Bacteroidetes in the metagenomic assemblies (Table S3) suggests this group has a substantial role in the BBD consortia. Marine Bacteroidetes are known for their ability to degrade complex polymers (Fernández-Gómez et al., 2013) and may thus play a critical role in the progression of BBD. Whether these Bacteroidetes are simply opportunistic in the degradation of coral tissue under a BBD mat or whether they contribute to the invasion of adjacent healthy tissue is yet to be determined. Recent work has demonstrated that benthic cyanobacterial mats in Caribbean coral reef ecosystems are stimulated by the localized degradation of organic matter in sediments (Brocke et al., 2015), further suggesting that microbial mat-forming filamentous cyanobacteria like *Roseofilum* require the release of nutrients by heterotrophs to proliferate. Here, we found that both Proteobacteria and Bacteroidetes in the five metagenomes carry glycoside hydrolase families for the degradation of starch/glycogen, oligosaccharides, and chitin (data not shown). Future metatranscriptomic studies may reveal which groups are most actively degrading complex carbon in the black band layer.

Akin to other cyanobacterial mats, a gradient of decreasing oxygen and increasing sulfide with depth in the black band layer has been detected (Glas et al., 2012), and the accumulation of sulfide may have an important role in BBD as sulfate reduction appears to be necessary for the initiation of the disease but not for the progression of already established BBD (Richardson et al., 2009; Brownell and Richardson, 2014). Previous investigations of key sulfur-cycling genes in BBD have identified dissimilatory sulfate reductase genes in *Desulfovibrio* (Bourne et al., 2011) and sulfur oxidation genes in Rhodobacteraceae (Bourne et al., 2013). However, the low levels and lack of diversity in sulfur oxidation genes associated with BBD imply that sulfide is not broken down, but rather accumulates beneath the mat (Bourne et al., 2013). These results are consistent with the current metagenomic study. Dissimilatory sulfate reduction genes (*dsrA*) were detected only in the two *Desulfovibrio* MAGs and not in the other major groups found in the black band consortium (Table S5). No sulfur oxidation genes of the *sox* pathway were detected in any of the five BBD metagenomes nor in the 26 MAGs, indicating that this pathway is likely rare in the black band layer, allowing the accumulation of sulfide during anaerobic phases of the diel cycle (Glas et al., 2012).

**Figure 4:**
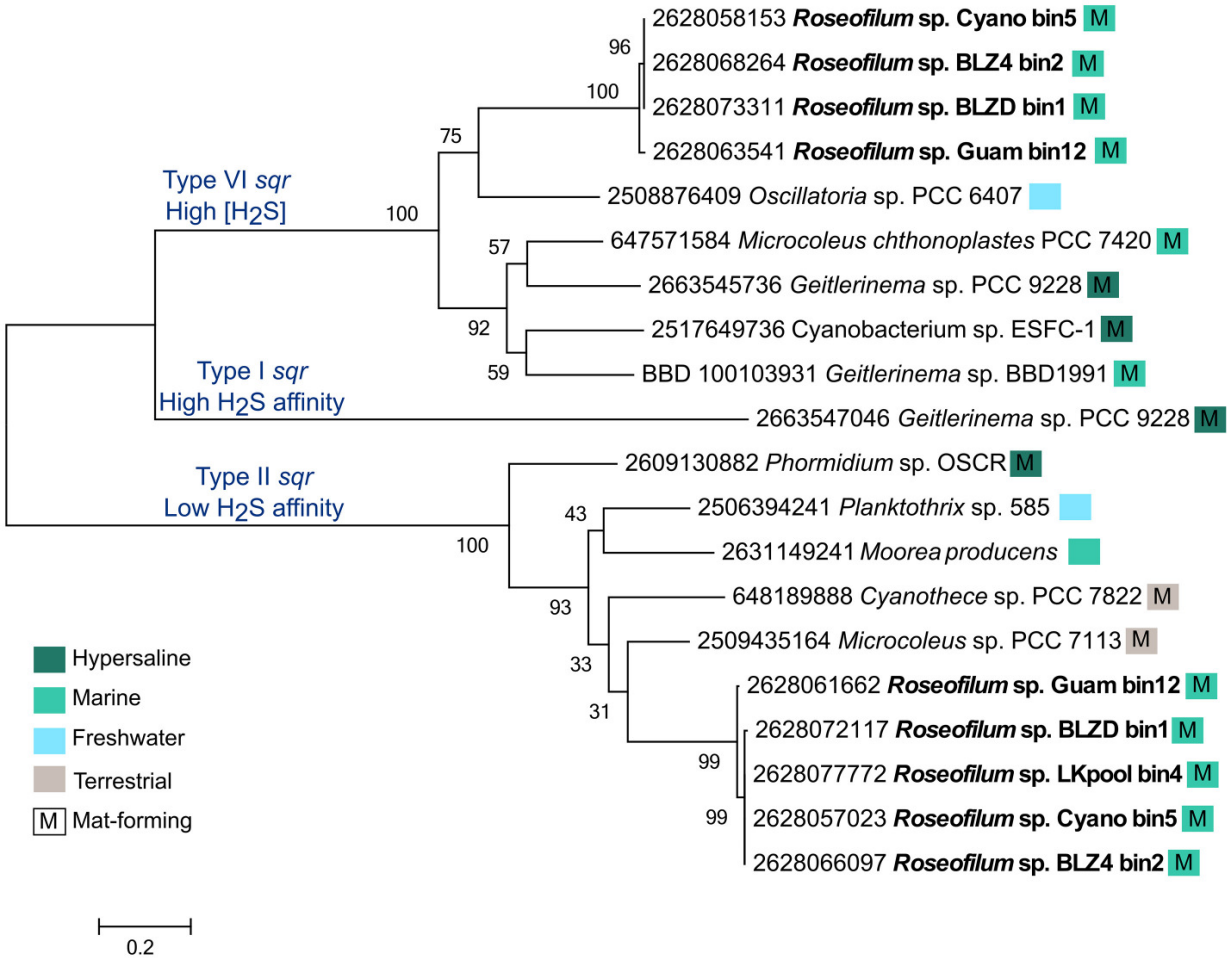
**Maximum likelihood tree of sulfide:quinone oxidoreductase (*sqr*) genes from filamentous cyanobacteria**. The five metagenome-assembled genomes of *Roseofilum* contained both Type VI and Type II *sqr* genes and are compared to *sqr* genes from other Oscillatoriales genomes. Branch labels are preceded by the IMG gene id and colored boxes indicate the habitat from which the cyanobacteria were isolated and whether or not it forms mats, based on publicly available metadata.

While few members of the disease consortium may be capable of oxidizing sulfide, it is possible that sulfide is detoxified through the action of sulfide:quinone oxidoreductase (*sqr*) genes. Each of the *Roseofilum* MAGs contains two unique copies of *sqr*, with the exception of the less complete Florida MAG (LKpool) that has only one assembled copy (Figure 4). The *Roseofilum* MAGs contain both Type VI and Type II *sqr* genes, which are adapted to high sulfide concentrations and low sulfide affinity, respectively (Marcia et al., 2010). The detoxification of sulfide appears to be a common feature in genomes of filamentous cyanobacteria that form mats, as this lifestyle promotes the growth of sulfate reducers and the sulfide they produce inhibits photosynthesis through the irreversible blockage of Photosystem I (Voorhies et al., 2012). The MAG of the *Geitlerinema* strain cultured from BBD (Den Uyl et al., 2016) also contains one copy of a Type VI *sqr*, that is about 58% similar to the Type VI *sqr* gene in the Caribbean *Roseofilum* MAGs (Figure 4). Neither *Roseofilum* nor *Geitlerinema* from black band contained the third type of *sqr* found in Cyanobacteria, Type I that has a high sulfide affinity and is used in anoxygenic photosynthesis in the filamentous cyanobacterium *Geitlerinema* sp. PCC 9228 (Grim and Dick, 2016). Single copies of *sqr* were also found in the unclassified gammaproteobacterium from the *Roseofilum* culture (IMG gene id 2628135161), in Bacteroidetes MAGs from Florida (2628091167), Belize (2628117824), and Guam (2628109795), and in the Rhodospirillales MAG from Belize (2628122119), indicating that the detoxification of sulfide is a common feature in members of the BBD polymicrobial consortium.

Metabolism of DMSP (dimethylsulfoniopropionate) has been implicated as an important process in the marine sulfur cycle, particularly in coral reefs, where DMSP functions as an osmolyte and is rapidly degraded by bacteria in the coral surface microbiome (Raina et al., 2009). No genes in the five metagenomes were annotated as DMSP lyases, which may reflect either the use of alternative pathways for DMSP degradation or the replacement of native coral commensals that would normally degrade DMSP with bacteria that are opportunistic generalists, lacking specialized adaptations to the coral epimicrobiome niche. In the light of extensive coral tissue death beneath the cyanobacterial mat and the lack of DMSP production genes in *Roseofilum*, it is unlikely that significant quantities of DMSP would be available in the black band layer, though this has not been directly measured.

### Production of secondary metabolites by black band disease consortia

**Figure 5:**
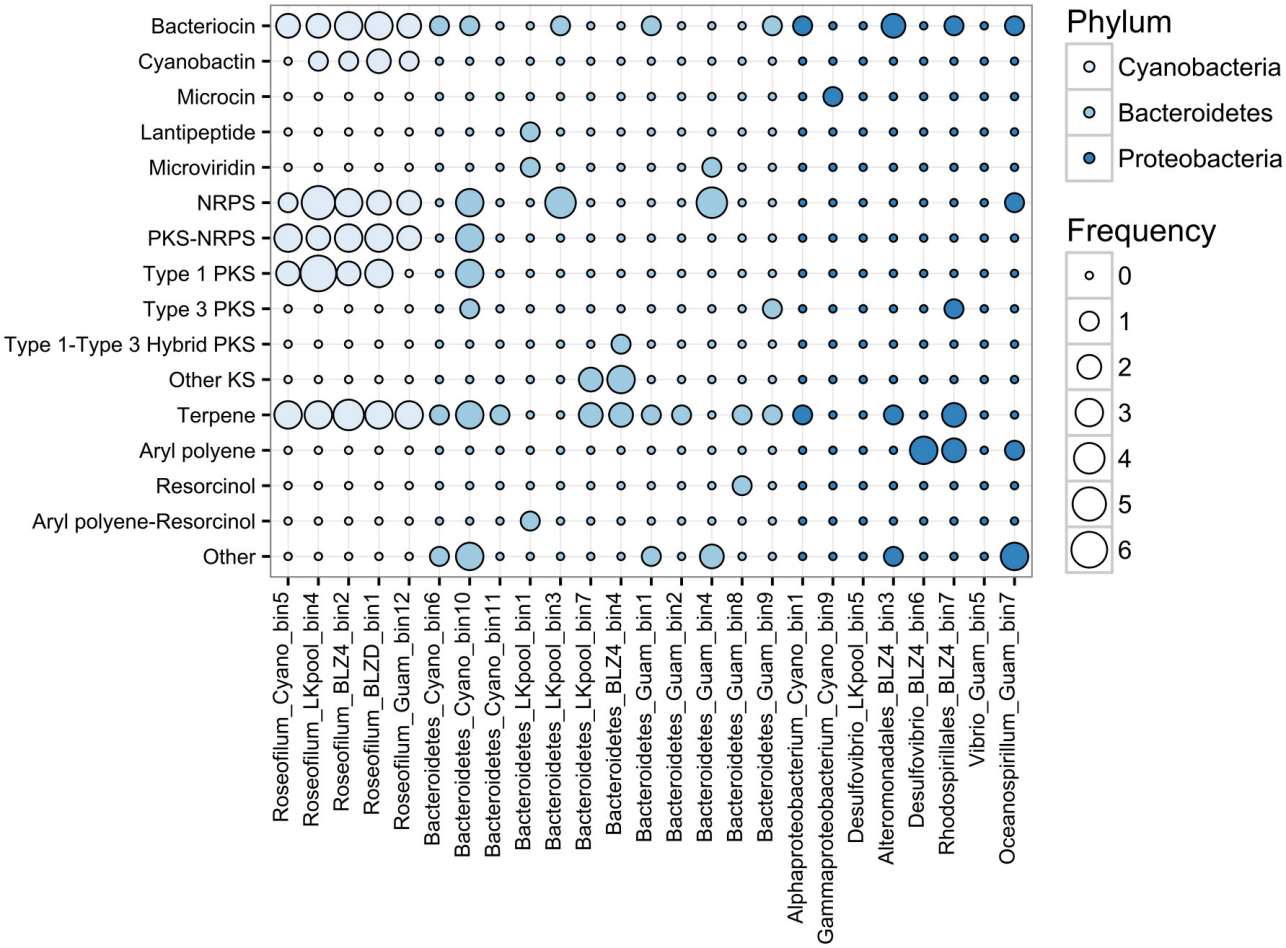
**Frequency of antibiotic and secondary metabolite biosynthetic clusters in binned genomes from Black Band Disease consortia**. Biosynthetic clusters were identified with AntiSMASH. Each column represents one metagenome-assembled genome and bubbles represent the relative abundance of biosynthetic clusters. NRPS, Non-ribosomal peptide synthase; PKS, polyketide synthase; Other KS, PKS other than type 1, 2, 3, or trans-AT.

Cyanobacteria are known for their ability to produce diverse natural products using non-ribosomal peptide synthetase (NRPS) and polyketide synthase (PKS) pathways, including toxins and siderophores (Leão et al., 2012; Shih et al., 2015). At least four unique NRPS/PKS biosynthetic clusters were detected per *Roseofilum* MAG, along with biosynthetic clusters for terpenes, bacteriocins, and cyanobactins (Figure 5). The abundance of biosynthetic clusters in the *Roseofilum* MAGs is consistent with the distribution of biosynthetic clusters in cyanobacterial genomes, as uncovered in a recent large-scale comparative genomics study (Shih et al., 2015). Natural products from marine cyanobacteria have been extensively explored for bioactive properties in the development of anti-inflammatory and anti-cancer drugs (Nunnery et al., 2010), though the intended purpose of these compounds from the perspective of cyanobacteria is potentially as grazing deterrents (Nagle and Paul, 1999), for UV protection (Sorrels et al., 2009) and allelopathy (Leão et al., 2012), or for interference in bacterial communication (quorum sensing inhibition; Dobretsov et al., 2010; Meyer et al., 2016). The *Roseofilum* MAGs contained the highest number of biosynthetic clusters, followed by Bacteroidetes MAGs, and the fewest clusters were detected in Proteobacteria MAGs. Two of the Proteobacteria MAGs (*Desulfovibrio* from the Belize *Orbicella* and *Vibrio* from the Guam *Goniopora*) had no detectable biosynthetic clusters for secondary metabolites. The role of secondary metabolites in the establishment and persistence of BBD consortia is an area of ongoing active research.

## Conclusions

The functional repertoire of BBD microbial communities highlights the role of the cyanobacterium *R. reptotaenium* as the engineer of the disease consortium through the production of organic carbon and nitrogen, as well as novel secondary metabolites. The *Roseofilum* MAGs recovered here from five different coral species and two different oceans contain a suite of both ribosome-dependent and non-ribosomal peptide and polyketide biosynthetic clusters, at copy numbers similar to or greater than other filamentous cyanobacteria (Shih et al., 2015). These secondary metabolites may be the key to understanding microbe-microbe interactions in the BBD microbial community, such as previously demonstrated for the role of lyngbic acid in inhibiting QS in *Vibrio* species (Meyer et al., 2016).

The genomic content of Bacteroidetes and Proteobacteria in BBD consortia suggests that heterotrophs fill the role of degrading and recycling organic matter within the mat, fueling the continued growth of *Roseofilum*. This dependence of *Roseofilum* on heterotrophic partners is consistent with the fact that filamentous cyanobacteria cannot be easily cultured axenically. However, the relationship between *Roseofilum* and its heterotrophic partners is not exclusively beneficial. For example, despite its potential for sulfide detoxification, *Roseofilum* migrates away from the build-up of sulfide within *in situ* BBD mats (Glas et al., 2012). In aerated cultures where *Desulfovibrio* is absent (and therefore not creating sulfide), *Roseofilum* can easily be overwhelmed by heterotrophic growth if the culture medium is too rich and must be transferred regularly to maintain active growth (*personal observation*). A similar accumulation of heterotrophic cells over time was documented in two different unicyanobacterial cultures of mats from Hot Lake, Washington (Cole et al., 2014).

The conservation of taxonomic groups in the minimal saltwater culture strongly suggests that once the disease consortium is established, the BBD microbial mat does not require the coral host tissue to thrive. This suggests that the pathogenesis of *Roseofilum* and other disease consortium members is contextual and has important implications for the mitigation of BBD in coral reef systems, primarily that if the conditions initiating the establishment of the microbial mat can be prevented, the disease can be thwarted.

## Author contributions

MT and VP conceived the project. MT, VP, and LR collected samples. JM performed the culturing and molecular work. All authors analyzed data and wrote the manuscript.

## Funding

JM is supported by the L'Oréal USA for Women in Science Fellowship and MT by the George E. Burch Fellowship in Theoretic Medicine and Affiliated Sciences from the Smithsonian Institution. This research was supported by Mote Marine Laboratory Protect Our Reefs grants POR 2012-1, POR 2013-2, and POR 2014-10 (MT, and VP).

### Conflict of interest statement

The authors declare that the research was conducted in the absence of any commercial or financial relationships that could be construed as a potential conflict of interest.

## Supplementary material

Supplementary Figure 1 **Comparison of (A)** the coverage (number of sequencing reads mapped to assembled metagenomes) of functional genes assigned to phyla in the non-axenic *Roseofilum* culture and four Black Band Disease mats and **(B)** the relative abundance of 16S amplicon sequences assigned to major bacterial phyla in the same samples.

Supplementary Figure 2 **NMDS plot of the Bray-Curtis similarity of functional profiles based on the presence or absence of genes assigned KEGG Orthology numbers in 26 binned genomes**. The phylum of each metagenome-assembled genome (MAG) is indicated by color and the metagenome origin of each MAG is indicated by the symbol.

Supplementary Figure 3 **Pairwise dot-plot comparisons of the contigs in *Roseofilum* metagenome-assembled genomes demonstrates high gene synteny among the four Caribbean-based samples and greater genome rearrangements in the Guam sample**.

Supplementary Table 1 **Summary of sample characteristics for 54 coral surface microbiomes and 1 Roseofilum culture used to assess bacterial community structure based on the V6 hypervariable region of 16S rRNA genes**.

Supplementary Table 2 **Characteristics of metagenome assemblies from Black Band Disease mats and cultured *Roseofilum reptotaenium***.

Supplementary Table 3 **Phylogenetic distribution of genes in metagenome assemblies from Black Band Disease mats and cultured *Roseofilum reptotaenium***.

Supplementary Table 4 **Differential represented SEED subsystems (Level 3) functions between three bacterial phyla in Black Band Disease metagenome-assembled genomes**.

Supplementary Table 5 **Genes associated with nitrogen and sulfur cycling in Black Band Disease metagenome-assembled genomes**.
