# Supplementary material for: Comparative Metagenomics of the Polymicrobial Black Band Disease of Corals
Source: Front Microbiol. 2017 Apr 18;8:618. doi: 10.3389/fmicb.2017.00618 (PMC5394123; doi:10.3389/fmicb.2017.00618)
Supplement: Supplementary Figure 1 — Comparison of (A) the coverage (number of sequencing reads mapped to assembled metagenomes) of functional genes assigned to phyla in the non-axenic Roseofilum culture and four Black Band Disease mats and (B) the relative abundance of 16S amplicon sequences assigned to major bacterial phyla in the same samples. [file Image1.PDF]

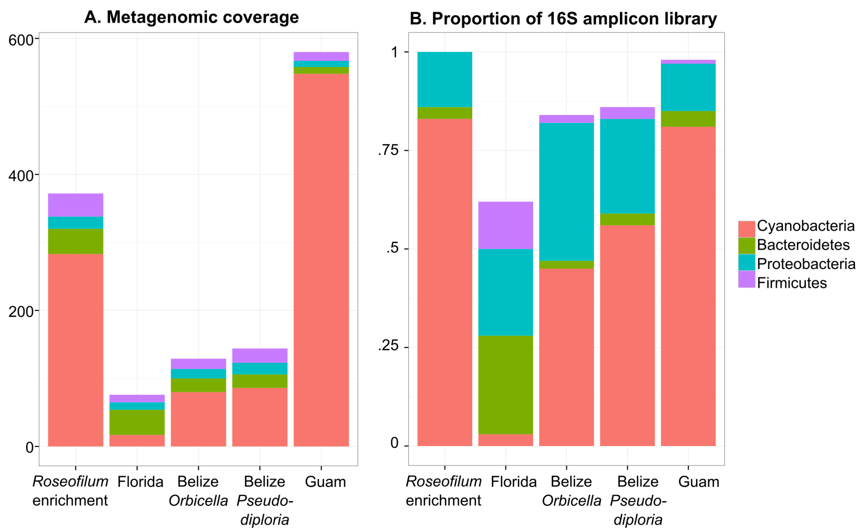

Figure S1. Comparison of A) the coverage (number of sequencing reads mapped to assembled metagenomes) of functional genes assigned to phyla in the non-axenic *Roseofilum* culture and four Black Band Disease mats and B) the relative abundance of 16S amplicon sequences assigned to major bacterial phyla in the same samples.
