# Supplementary figures and images for: Comparative Metagenomics of the Polymicrobial Black Band Disease of Corals
Source: Front Microbiol. 2017 Apr 18;8:618. doi: 10.3389/fmicb.2017.00618 (PMC5394123; doi:10.3389/fmicb.2017.00618)

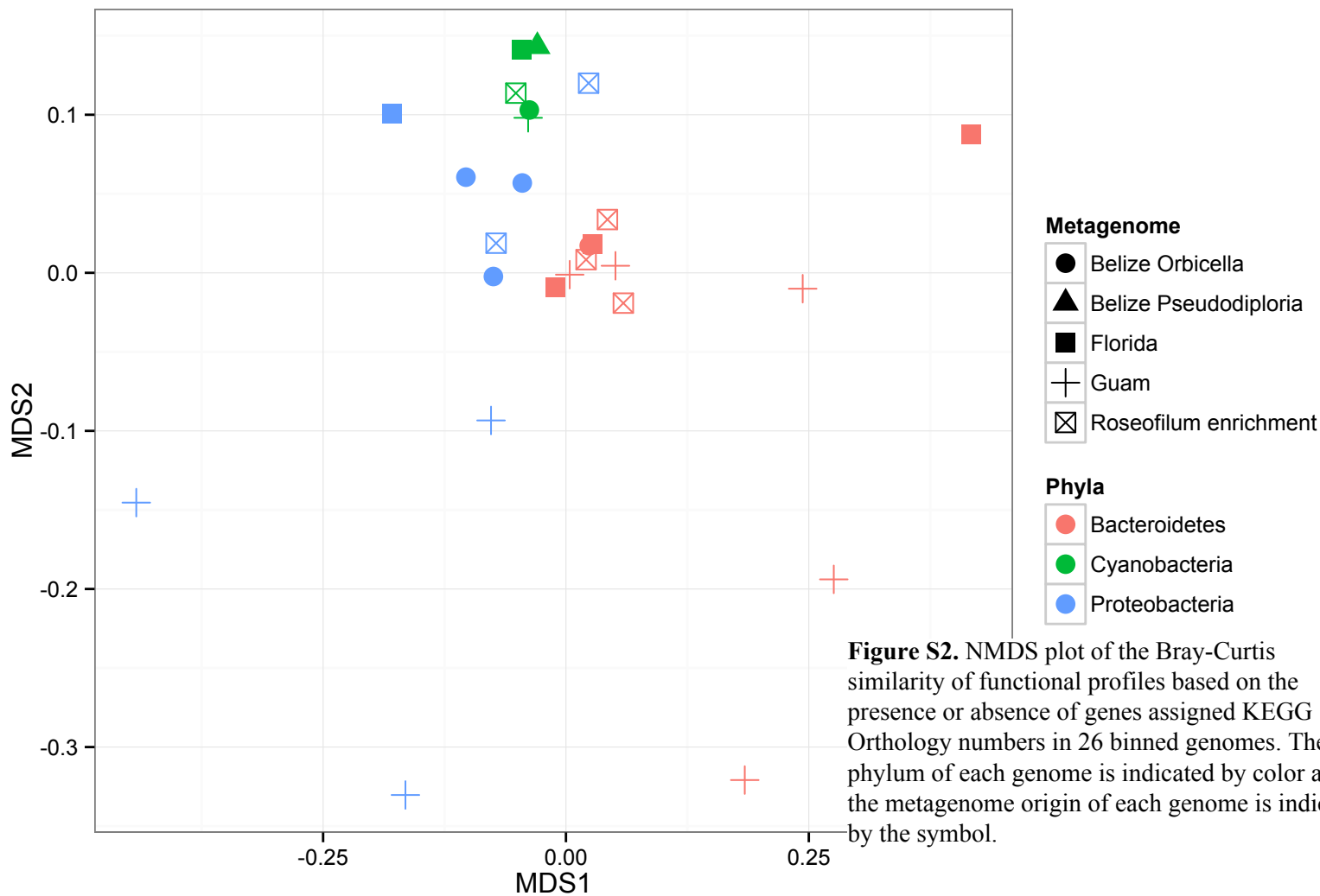

Supplement: Supplementary Figure 2 — NMDS plot of the Bray-Curtis similarity of functional profiles based on the presence or absence of genes assigned KEGG Orthology numbers in 26 binned genomes. The phylum of each metagenome-assembled genome (MAG) is indicated by color and the metagenome origin of each MAG is indicated by the symbol. [file Image2.PDF]
