# Supplementary material for: Comparative Metagenomics of the Polymicrobial Black Band Disease of Corals
Source: Front Microbiol. 2017 Apr 18;8:618. doi: 10.3389/fmicb.2017.00618 (PMC5394123; doi:10.3389/fmicb.2017.00618)
Supplement: Supplementary Figure 3 — Pairwise dot-plot comparisons of the contigs in Roseofilum metagenome-assembled genomes demonstrates high gene synteny among the four Caribbean-based samples and greater genome rearrangements in the Guam sample. [file Image3.pdf]

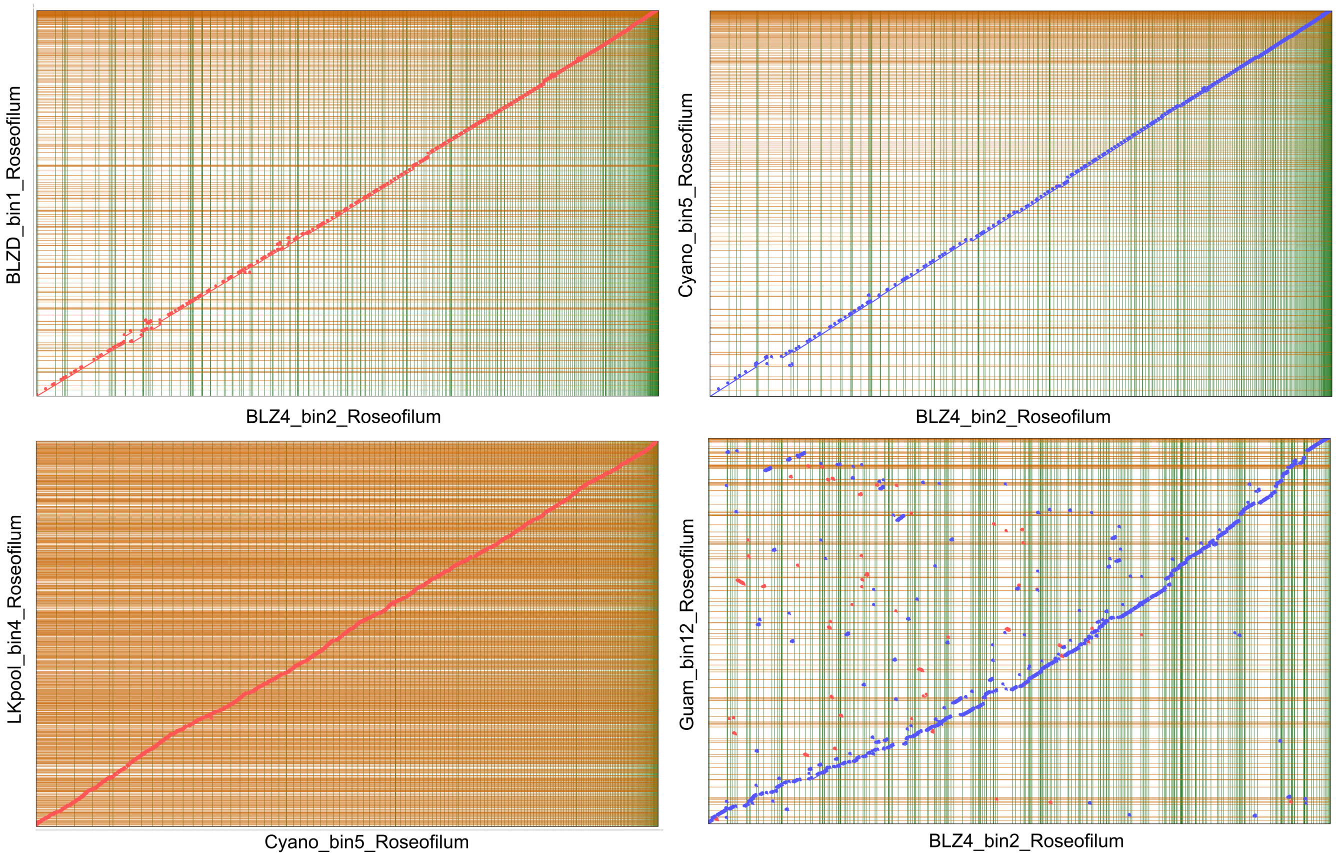

Figure S3. Pairwise dot-plot comparisons of the contigs in *Roseofilum* genomes demonstrates high gene synteny among the four Caribbean-based samples and greater genome rearrangements in the Guam sample.
