# Supplementary material for: Comparative Metagenomics of the Polymicrobial Black Band Disease of Corals
Source: Front Microbiol. 2017 Apr 18;8:618. doi: 10.3389/fmicb.2017.00618 (PMC5394123; doi:10.3389/fmicb.2017.00618)
Supplement: Supplementary Table 1 — Summary of sample characteristics for 54 coral surface microbiomes and 1 Roseofilum culture used to assess bacterial community structure based on the V6 hypervariable region of 16S rRNA genes. [file Table1.PDF]

Table S1. Summary of sample characteristics for 54 coral surface microbiomes and 1 *Roseofilum* culture used to assess bacterial community structure based on the V6 hypervariable region of 16S rRNA genes.

Sample	Health	Location	Coral Host	Collection Date	Sequences
A46BB	Black Band	Belize[1]	*Orbicella annularis*	Aug-14	374,772
A50BB	Black Band	Belize	*Orbicella faveolata*	Aug-14	197,198
BLZ2	Black Band	Belize	*Orbicella annularis*	Jul-13	126,593
BLZ4*	Black Band	Belize	*Orbicella annularis*	Jul-13	103,407
BLZ5	Black Band	Belize	*Orbicella annularis*	Jul-13	85,287
J32BB	Black Band	Belize	*Orbicella annularis*	Jul-13	232,079
MNC	Black Band	Belize	*Orbicella annularis*	Aug-14	264,985
BOF	Black Band	Belize	*Orbicella faveolata*	Aug-14	475,847
DCL	Black Band	Belize	*Pseudodiploria clivosa*	Aug-14	9,498
BPS	Black Band	Belize	*Pseudodiploria strigosa*	Aug-14	222,362
DBB	Black Band	Belize	*Pseudodiploria strigosa*	Aug-14	210,565
A28NC	Healthy Coral	Belize	*Orbicella annularis*	Aug-14	19,215
A33NC	Healthy Coral	Belize	*Orbicella annularis*	Aug-14	86,598
A44NC	Healthy Coral	Belize	*Orbicella annularis*	Aug-14	40,109
F32NC	Healthy Coral	Belize	*Orbicella annularis*	Feb-13	582,387
M40FT	Healthy Coral	Belize	*Orbicella annularis*	Aug-14	76,764
M47FT	Healthy Coral	Belize	*Orbicella annularis*	Aug-14	95,249
M57FT	Healthy Coral	Belize	*Orbicella annularis*	Aug-14	92,464
M61FTA	Healthy Coral	Belize	*Orbicella annularis*	Aug-14	97,566
M61FTB	Healthy Coral	Belize	*Orbicella annularis*	Aug-14	279,181
M70FT	Healthy Coral	Belize	*Orbicella annularis*	Aug-14	438,975
A46NC	Healthy Tissue	Belize	*Orbicella annularis*	Aug-14	27,814
A50NC	Healthy Tissue	Belize	*Orbicella faveolata*	Aug-14	184,473
F28NC	Healthy Tissue	Belize	*Orbicella annularis*	Feb-13	605,049
F29NC	Healthy Tissue	Belize	*Orbicella annularis*	Feb-13	749,884
F33NC	Healthy Tissue	Belize	*Orbicella annularis*	Feb-13	300,367
F44NC	Healthy Tissue	Belize	*Orbicella annularis*	Feb-13	476,809
F50NC	Healthy Tissue	Belize	*Orbicella faveolata*	Feb-13	486,621
J32NC	Healthy Tissue	Belize	*Orbicella annularis*	Jul-13	6,577
J44HE	Healthy Tissue	Belize	*Orbicella annularis*	Jul-13	78,016
J44NC	Healthy Tissue	Belize	*Orbicella annularis*	Jul-13	33,238
MBB	Healthy Tissue	Belize	*Orbicella annularis*	Aug-14	82,393
DNC	Healthy Tissue	Belize	*Pseudodiploria strigosa*	Aug-14	218,292
BLZD*	Black Band	Belize	*Pseudodiploria strigosa*	Feb-13	155,453
FL1	Black Band	Florida	*Montastraea cavernosa*	May-13	115,256
FL2	Black Band	Florida[2]	*Montastraea cavernosa*	May-13	408,160
FL4	Black Band	Florida	*Montastraea cavernosa*	May-13	335,680
FL5	Black Band	Florida	*Montastraea cavernosa*	May-13	57,362
LK1[+]	Black Band	Florida	*Montastraea cavernosa*	Jun-13	244,667
LK2[+]	Black Band	Florida	*Orbicella faveolata*	Jun-13	158,302
LK4[+]	Black Band	Florida	*Orbicella faveolata*	Jun-13	219,775
Cyano*	Enrichment culture	Florida	*Montastraea cavernosa*	Apr-14	524,581
FLA	Black Band	Florida	*Montastraea cavernosa*	Apr-14	229,768
FLB	Black Band	Florida	*Montastraea cavernosa*	Apr-14	152,408
FLC	Black Band	Florida	*Montastraea cavernosa*	Apr-14	342,213
FLA1	Healthy Tissue	Florida	*Montastraea cavernosa*	Apr-14	159,649
FLA2	Healthy Tissue	Florida	*Montastraea cavernosa*	Apr-14	133,858
FLB1	Healthy Tissue	Florida	*Montastraea cavernosa*	Apr-14	70,947

FLB2	Healthy Tissue	Florida	*Montastraea cavernosa*	Apr-14	185,161
FLB3	Healthy Tissue	Florida	*Montastraea cavernosa*	Apr-14	356,762
FLC1	Healthy Tissue	Florida	*Montastraea cavernosa*	Apr-14	136,954
FLC2	Healthy Tissue	Florida	*Montastraea cavernosa*	Apr-14	357,678
FLC3	Healthy Tissue	Florida	*Montastraea cavernosa*	Apr-14	127,307
Guam1*	Black Band	Guam[3]	*Goniopora fruticosa*	Jun-14	335,373
Guam2	Black Band	Guam	*Goniopora fruticosa*	Jun-14	280,831

* Metagenomic sequencing also performed on these samples.
[+] Florida samples pooled for metagenomic sequencing.
[1] Carrie Bow Cay, Belize: 16.797 N, 88.084 W
[2] Looe Key Reef, Florida, USA: 24.548 N, 81.406 W
[3] Luminao Reef Flat, Guam: 13.464 N, 144.644 E
