# Supplementary material for: Comparative Metagenomics of the Polymicrobial Black Band Disease of Corals
Source: Front Microbiol. 2017 Apr 18;8:618. doi: 10.3389/fmicb.2017.00618 (PMC5394123; doi:10.3389/fmicb.2017.00618)
Supplement: Supplementary Table 2 — Characteristics of metagenome assemblies from Black Band Disease mats and cultured Roseofilum reptotaenium. [file Table2.PDF]

Table S2. Characteristics of metagenomes from Black Band Disease mats and cultured *Roseofilum reptotaenium*.

**Metagenome locus tag**	**Cyano**	**LKpool**	**BLZ4**	**BLZD**	**Guam**
**IMG Genome ID**	3300003272	3300003311	3300003317	3300003641	3300003309
**Collection location**	Looe Key Reef, Florida	Looe Key Reef, Florida	Carrie Bow Cay, Belize	Carrie Bow Cay, Belize	Luminao Reef Flat, Guam
**Collection date**	April 2014	June 2013	July 2013	Feb 2013	June 2014
**Coral host**	*Roseofilum* culture, isolated from *Montastraea cavernosa*	pooled: *Montastraea cavernosa* and *Orbicella faveolata*	*Orbicella annularis*	*Pseudodiploria strigosa*	*Goniopora fruticosa*
**Read pairs**	34,974,648	32,180,161	34,193,363	16,853,081	44,199,247
**SRA Accession**	SRX1017628	SRX1017632	SRX894068	SRX894049	SRX1017629
**Scaffolds**	62,162	186,345	524,359	56,139	151,976
**Total length of contigs (bp)**	117,262,816	124,960,495	279,467,519	34,028,000	113,479,370
**Protein coding genes**	145,980	238,319	612,757	69,548	207,250
**Proportion of reads mapped to assembled scaffolds**	92%	61%	72%	41%	71%
**Estimated sequencing coverage**	90%	85%	87%	99%	84%
**Estimated number of genomes in metagenome**	19 - 22	14 - 17	6 -19	2 - 4	7 - 24
**Binned genomes**	6	5	5	1	9
