# Supplementary material for: Comparative Metagenomics of the Polymicrobial Black Band Disease of Corals
Source: Front Microbiol. 2017 Apr 18;8:618. doi: 10.3389/fmicb.2017.00618 (PMC5394123; doi:10.3389/fmicb.2017.00618)
Supplement: Supplementary Table 3 — Phylogenetic distribution of genes in metagenome assemblies from Black Band Disease mats and cultured Roseofilum reptotaenium. [file Table3.PDF]

Table S3. Phylogenetic distribution of genes in metagenome assemblies from Black Band Disease mats and cultured Roseofilum reptotaenium.

Domain	Phylum	Cyano 3300003272 Gene Count	Cyano 3300003272 %	Guam 3300003309 Gene Count	Guam 3300003309 %	LKpool 3300003311 Gene Count	LKpool 3300003311 %	BLZ4 3300003317 Gene Count	BLZ4 3300003317 %	BLZD 3300003641 Gene Count	BLZD 3300003641 %
Bacteria	Bacteroidetes	20329	13.93	25494	12.3	30784	12.92	17397	2.84	5904	8.49
Bacteria	Proteobacteria	18877	12.93	25163	12.14	15006	6.3	29159	4.76	3920	5.64
Bacteria	Cyanobacteria	4460	3.06	3679	1.78	4119	1.73	5939	0.97	2778	3.99
Bacteria	Firmicutes	3086	2.11	3922	1.89	9370	3.93	11437	1.87	1858	2.67
Bacteria	Actinobacteria	2174	1.49	762	0.37	1056	0.44	3356	0.55	408	0.59
Bacteria	Cloacimonetes	60	0.04	124	0.06	106	0.04	199	0.03	371	0.53
Bacteria	Verrucomicrobia	1927	1.32	277	0.13	390	0.16	1206	0.2	189	0.27
Archaea	Euryarchaeota	612	0.42	427	0.21	553	0.23	2254	0.37	166	0.24
Bacteria	Spirochaetes	433	0.3	484	0.23	766	0.32	1368	0.22	155	0.22
Bacteria	Lentisphaerae	306	0.21	26	0.01	625	0.26	617	0.1	143	0.21
Bacteria	Planctomycetes	12015	8.23	255	0.12	418	0.18	862	0.14	140	0.2
Bacteria	Fusobacteria	66	0.05	145	0.07	294	0.12	474	0.08	72	0.1
Bacteria	Chloroflexi	2136	1.46	141	0.07	160	0.07	474	0.08	68	0.1
Bacteria	Chlorobi	148	0.1	180	0.09	187	0.08	233	0.04	53	0.08
Bacteria	Acidobacteria	474	0.32	110	0.05	119	0.05	323	0.05	39	0.06
Bacteria	Ignavibacteriae	106	0.07	839	0.4	153	0.06	156	0.03	39	0.06
Bacteria	Thermotogae	50	0.03	39	0.02	85	0.04	237	0.04	34	0.05
Bacteria	Aquificae	70	0.05	54	0.03	79	0.03	308	0.05	26	0.04
Bacteria	Deinococcus-Thermus	389	0.27	85	0.04	73	0.03	247	0.04	31	0.04
Bacteria	Aminicenantes	112	0.08	105	0.05	89	0.04	113	0.02	26	0.04
Archaea	Crenarchaeota	69	0.05	48	0.02	57	0.02	564	0.09	19	0.03
Bacteria	Chlamydiae	65	0.04	42	0.02	37	0.02	241	0.04	19	0.03
Bacteria	Synergistetes	43	0.03	40	0.02	66	0.03	99	0.02	24	0.03
Bacteria	Marinimicrobia	56	0.04	57	0.03	85	0.04	125	0.02	18	0.03
Bacteria	Caldithrixae	62	0.04	100	0.05	80	0.03	134	0.02	21	0.03
Bacteria	Tenericutes	38	0.03	53	0.03	61	0.03	539	0.09	14	0.02
Bacteria	Deferribacteres	39	0.03	43	0.02	74	0.03	163	0.03	15	0.02
Bacteria	Atribacteria	29	0.02	46	0.02	79	0.03	53	0.01	11	0.02
Bacteria	Latescibacteria	88	0.06	47	0.02	78	0.03	79	0.01	16	0.02
Bacteria	unclassified	29	0.02	46	0.02	46	0.02	56	0.01	11	0.02
Bacteria	Parcubacteria	9	0.01	11	0.01	16	0.01	102	0.02	10	0.01
Bacteria	Thermodesulfobacteria	31	0.02	14	0.01	27	0.01	94	0.02	4	0.01
Bacteria	Nitrospirae	43	0.03	21	0.01	27	0.01	51	0.01	6	0.01
Bacteria	Hydrogenedentes	33	0.02	9	0	21	0.01	36	0.01	4	0.01
Bacteria	Fibrobacteres	25	0.02	21	0.01	39	0.02	39	0.01	8	0.01
Bacteria	Gemmatimonadetes	93	0.06	19	0.01	20	0.01	64	0.01	8	0.01
Bacteria	BRC1	73	0.05	12	0.01	27	0.01	34	0.01	8	0.01
Bacteria	Poribacteria	48	0.03	9	0	15	0.01	34	0.01	9	0.01
Bacteria	Aerophobetes	33	0.02	17	0.01	18	0.01	22	0	8	0.01
Bacteria	Armatimonadetes	69	0.05	10	0	10	0	22	0	4	0.01
Archaea	Thaumarchaeota	11	0.01	10	0	10	0	106	0.02	2	0
Bacteria	Gracilibacteria	4	0	24	0.01	12	0.01	119	0.02	0	0
Archaea	Aigarchaeota	6	0	11	0.01	3	0	40	0.01	0	0
Bacteria	Microgenomates	4	0	1	0	2	0	47	0.01	0	0
Bacteria	Chrysiogenetes	18	0.01	9	0	9	0	33	0.01	1	0
Bacteria	Dictyoglomi	15	0.01	8	0	14	0.01	50	0.01	0	0
Archaea	Candidatus Korarchaeota	7	0	4	0	5	0	58	0.01	2	0
Bacteria	Nitrospinae	39	0.03	9	0	25	0.01	39	0.01	3	0
Bacteria	Calescamantes	7	0	2	0	4	0	22	0	0	0
Bacteria	Fervidibacteria	27	0.02	5	0	6	0	16	0	1	0
Bacteria	Omnitrophica	34	0.02	8	0	11	0	24	0	2	0
Archaea	Diapherotrites	5	0	4	0	4	0	21	0	0	0
Bacteria	Caldiserica	4	0	1	0	5	0	22	0	0	0
Bacteria	Candidatus Saccharibacteria	4	0	0	0	4	0	28	0	0	0
Bacteria	Elusimicrobia	3	0	2	0	8	0	5	0	1	0
Bacteria	Acetothermia	4	0	4	0	7	0	2	0	1	0
Bacteria	EM3	2	0	0	0	0	0	1	0	0	0
Bacteria	PER	1	0	4	0	3	0	30	0	2	0
Bacteria	WS1	6	0	2	0	1	0	3	0	2	0
