# Supplementary material for: Comparative Metagenomics of the Polymicrobial Black Band Disease of Corals
Source: Front Microbiol. 2017 Apr 18;8:618. doi: 10.3389/fmicb.2017.00618 (PMC5394123; doi:10.3389/fmicb.2017.00618)
Supplement: Supplementary Table 5 — Genes associated with nitrogen and sulfur cycling in Black Band Disease metagenome-assembled genomes. [file Table5.PDF]

Table S5. Genes associated with nitrogen and sulfur cycling in Black Band Disease metagenome-assembled genomes.

IMG gene_oid	IMG Locus Tag	Gene Product Name	IMG Genome ID	Genome Name
2628137143	Ga0079951_10908	cyanophycin synthetase	2627853583	Cyano_bin9_Gammaproteobacteria Ga0079951
2628137144	Ga0079951_10909	cyanophycin synthetase	2627853583	Cyano_bin9_Gammaproteobacteria Ga0079951
2628137703	Ga0079951_11613	cyanophycinase	2627853583	Cyano_bin9_Gammaproteobacteria Ga0079951
2628137704	Ga0079951_11614	cyanophycin synthetase	2627853583	Cyano_bin9_Gammaproteobacteria Ga0079951
2628068922	Ga0079929_112012	cyanophycin synthetase	2627853561	Roseofilum sp. BLZ4_bin2 Ga0079929
2628067756	Ga0079929_10588	cyanophycinase	2627853561	Roseofilum sp. BLZ4_bin2 Ga0079929
2628068923	Ga0079929_112013	cyanophycinase	2627853561	Roseofilum sp. BLZ4_bin2 Ga0079929
2628072855	Ga0079930_105840	cyanophycin synthetase	2627853562	Roseofilum sp. BLZD_bin1 Ga0079930
2628072856	Ga0079930_105841	cyanophycinase	2627853562	Roseofilum sp. BLZD_bin1 Ga0079930
2628072760	Ga0079930_105511	cyanophycinase	2627853562	Roseofilum sp. BLZD_bin1 Ga0079930
2628058810	Ga0079927_105340	cyanophycin synthetase	2627853559	Roseofilum sp. Cyano_bin5 Ga0079927
2628059528	Ga0079927_10888	cyanophycinase	2627853559	Roseofilum sp. Cyano_bin5 Ga0079927
2628058811	Ga0079927_105341	cyanophycinase	2627853559	Roseofilum sp. Cyano_bin5 Ga0079927
2628062588	Ga0079928_102436	cyanophycin synthetase	2627853560	Roseofilum sp. Guam_bin12 Ga0079928
2628062589	Ga0079928_102437	cyanophycinase	2627853560	Roseofilum sp. Guam_bin12 Ga0079928
2628062244	Ga0079928_101985	cyanophycinase	2627853560	Roseofilum sp. Guam_bin12 Ga0079928
2628077039	Ga0079931_13262	cyanophycinase	2627853563	Roseofilum sp. LKpool_bin4 Ga0079931
2628062393	Ga0079928_102162	Mo-nitrogenase MoFe protein subunit NifK	2627853560	Roseofilum sp. Guam_bin12 Ga0079928
2628060320	Ga0079927_117712	Mo-nitrogenase MoFe protein subunit NifK	2627853559	Roseofilum sp. Cyano_bin5 Ga0079927
2628129200	Ga0079949_100635	Mo-nitrogenase iron protein subunit NifH	2627853581	Alteromonadales bin3 BLZ4 Ga0079949
2628075613	Ga0079931_10724	Mo-nitrogenase MoFe protein subunit NifK	2627853563	Roseofilum sp. LKpool_bin4 Ga0079931
2628071631	Ga0079930_102629	Mo-nitrogenase iron protein subunit NifH	2627853562	Roseofilum sp. BLZD_bin1 Ga0079930
2628062394	Ga0079928_102163	nitrogenase molybdenum-iron protein alpha chain	2627853560	Roseofilum sp. Guam_bin12 Ga0079928
2628071635	Ga0079930_102633	Mo-nitrogenase MoFe protein subunit NifK	2627853562	Roseofilum sp. BLZD_bin1 Ga0079930
2628071634	Ga0079930_102632	nitrogenase molybdenum-iron protein alpha chain	2627853562	Roseofilum sp. BLZD_bin1 Ga0079930

2628124723	Ga0079947_14533	Mo-nitrogenase iron protein subunit NifH	2627853579	Rhodospirillales bin7 BLZ4 Ga0079947
2628060321	Ga0079927_117713	nitrogenase molybdenum-iron protein alpha chain	2627853559	Roseofilum sp. Cyano_bin5 Ga0079927
2628060324	Ga0079927_117716	Mo-nitrogenase iron protein subunit NifH	2627853559	Roseofilum sp. Cyano_bin5 Ga0079927
2628124724	Ga0079947_14534	Nitrogenase component 1 type Oxidoreductase	2627853579	Rhodospirillales bin7 BLZ4 Ga0079947
2628131269	Ga0079949_112512	Mo-nitrogenase MoFe protein subunit NifK	2627853581	Alteromonadales bin3 BLZ4 Ga0079949
2628125228	Ga0079947_16012	Mo-nitrogenase MoFe protein subunit NifK	2627853579	Rhodospirillales bin7 BLZ4 Ga0079947
2628131270	Ga0079949_112513	Mo-nitrogenase MoFe protein subunit NifD precursor	2627853581	Alteromonadales bin3 BLZ4 Ga0079949
2628066997	Ga0079929_10325	nitrogenase molybdenum-iron protein alpha chain	2627853561	Roseofilum sp. BLZ4_bin2 Ga0079929
2628075612	Ga0079931_10723	nitrogenase molybdenum-iron protein alpha chain	2627853563	Roseofilum sp. LKpool_bin4 Ga0079931
2628067000	Ga0079929_10328	Mo-nitrogenase iron protein subunit NifH	2627853561	Roseofilum sp. BLZ4_bin2 Ga0079929
2628062390	Ga0079928_102159	nitrogenase molybdenum-iron protein NifN	2627853560	Roseofilum sp. Guam_bin12 Ga0079928
2628125227	Ga0079947_16011	Nitrogenase component 1 type Oxidoreductase	2627853579	Rhodospirillales bin7 BLZ4 Ga0079947
2628066996	Ga0079929_10324	Mo-nitrogenase MoFe protein subunit NifK	2627853561	Roseofilum sp. BLZ4_bin2 Ga0079929
2628083846	Ga0079933_14002	dissimilatory adenylylsulfate reductase beta subunit	2627853565	Desulfovibrio bin6 BLZ4 Ga0079933
2628080331	Ga0079932_12942	dissimilatory adenylylsulfate reductase beta subunit	2627853564	LKpool_bin5_Desulfovibrio Ga0079932
2628083845	Ga0079933_14001	dissimilatory adenylylsulfate reductase alpha subunit precursor	2627853565	Desulfovibrio bin6 BLZ4 Ga0079933
2628080330	Ga0079932_12941	dissimilatory adenylylsulfate reductase alpha subunit precursor	2627853564	LKpool_bin5_Desulfovibrio Ga0079932
